# *Mycobacterium ulcerans* mycolactones-fungi crosstalking

**DOI:** 10.1038/s41598-019-39927-3

**Published:** 2019-02-28

**Authors:** Nassim Hammoudi, Carole Cassagne, Nicholas Armstrong, Stéphane Ranque, Bernard Henrissat, Michel Drancourt, Amar Bouam

**Affiliations:** 1 Aix-Marseille Univ, IRD, MEPHI, IHU Méditerranée Infection, Marseille, France; 2 Aix Marseille Univ, IRD, AP-HM, SSA, VITROME, IHU Méditerranée Infection, Marseille, France; 3 Architecture et Fonction des Macromolécules Biologiques, CNRS, Aix-Marseille Université, Marseille, France

## Abstract

The opportunistic pathogen *Mycobacterium ulcerans*, which is responsible for Buruli ulcer, synthesizes a series of plasmid-encoded macrolide exotoxins termed mycolactones. These toxins destabilize cell membranes and induce apoptosis-associated pleiotropic effects including tissue destruction, analgesic and anti-inflammatory effects. Despite its medical interest, *M. ulcerans* is primarily an environmental mycobacterium and the primary functions of mycolactones in the natural ecosystems are unknown. High throughput biochemical profiling findings suggested that *M. ulcerans* may interact with fungi. Here, we report that semi-purified and purified mycolactones significantly enhance spore germination of *Scedosporium apiospermum, Fusarium equiseti* and *Mucor circinelloides*; and that *M. ulcerans* mycolactones significantly attract colonies of *M. circinelloides* whereas no significant effect was observed on *S. apiospermum* and *F. equiseti*. These experimental results suggest that mycolactones exhibit a chemoattractant activity independent of their cytotoxicity. In natural ecosystems, *M. ulcerans* mycolactones may act as spore germination inducers and chemoattractants for some fungi, suggesting a novel role for this unique class of mycobacterial toxins in natural ecosystems.

## Introduction

Mycolactones are a series of complex macrolide exotoxins whose plasmid-encoded synthesis is specific to the *Mycobacterium marinum* group of non-tuberculous mycobacteria^1^. Mycolactones are hybrid polyketides comprising a conserved lactone core decorated with a variable fatty acid side chain. Six naturally occurring mycolactone types have been named mycolactones A/B, C, D, E, F and G^2^. These mycolactones are produced by a group of closely related mycobacteria including *M. marinum*, *Mycobacterium ulcerans* and *M. ulcerans* subsp. *shinshuense*, *Mycobacterium pseudoshottsii* and *Mycobacterium liflandii*^3–8^. In addition to its natural production, mycolactone A/B has also undergone total synthesis^1,9^.

In Buruli ulcer patients and animal models, mycolactones induce apoptosis in a large variety of cells including Schwann cells^10^ by activating the pro-apoptotic regulator Bim^11^. Accordingly, intradermic injection of mycolactones in laboratory animals or natural inoculation by still unknown vectors in human patients both cause extensive and disabling cutaneous and subcutaneous lesions referred to as Buruli ulcer in patients^2^. Mycolactones further exhibit immunosuppressive effects by inhibiting the T cells Sec61 protein^12,13^ and binding of mycolactone A/B to the neuronal angiotensin II type 2 receptors triggers potassium-dependent neuron hyperpolarization and analgesic effects^14^. Each of these features has been observed in patients affected with Buruli ulcer^2^.

Buruli ulcer is a non-contagious mycobacteriosis, thus human infection is a dead-end in *M. ulcerans* life cycle^2^. *M. ulcerans* reservoir is environmental in still poorly defined ecosystems near stagnant water. Its DNA has been detected in plants, moss, water bugs, mosquitoes, snails, fishes, amphibians and small mammals^2^. Acquisition and conservation of complex mycolactone synthesis machinery contrasts with the global genome reduction that characterizes *M. ulcerans* evolution^15^. The primary roles of mycolactones in the natural ecosystems remain unknown. In line with its aquatic environment, a previous high throughput biochemical profiling study revealed the potential for specific interactions between *M. ulcerans* and mollusks, bacterial consortia, algae and fungi^16^. We hypothesized that mycolactones may play a role in these interactions and therefore tested possible interactions between mycolactones and environmental fungi. We found that mycolactones exhibit a chemoattractant and spore germination enhancer effect on three environmental fungi that we investigated.

## Methods

### *M. ulcerans* and fungal strains

*M. ulcerans* CU001 was cultured into a BLS3 laboratory on Middlebrook 7H10 agar medium supplemented with 10% (v/v) of oleic acid/albumin/dextrose/catalase (OADC) (Becton Dickinson, Sparks, MD, USA) under an aerobic atmosphere at 30 °C. The identity of colonies used for experiments was confirmed by the detection of IS2404, IS2606 and KR-B using a multiplex real time PCR as previously described^17^. Clinical isolates of three fungi *Mucor circinelloides, Scedosporium apiospermum* and *Fusarium equiseti*, were cultured on Sabouraud gentamicin chloramphenicol agar (SGC, Oxoid, Dardilly, France) for 72 h at 30 °C in an ambiant atmosphere. The fungal isolates were identified by both matrix-assisted laser desorption/ionization time-of-flight (MALDI-TOF) mass spectrometry and DNA sequencing as previously described^18^. Fungal spores were harvested from 72 hour-culture on SGC agar medium by adding 2 mL of Sabouraud liquid medium on a culture of each fungal species and gently pipetting the liquid medium without touching the mycelia. Then 2 mL of liquid medium were pipetted and placed into a tube containing 6 mL of Sabouraud liquid medium and vortexed.

### Mycolactone preparation and biological activity tests

Mycolactones were prepared from *M. ulcerans* CU001 cells as previously described^5,19^. Briefly, six-week-old colonies were inactivated at 90 °C for one hour. Colonies were centrifuged at 3,500 g for 15 min and the pellet suspended in chloroform-methanol (2:1, v/v). Folch’s extraction was performed by adding 0.2 volumes of water. The lower organic phase containing total lipids was collected, dried at 40 °C under a stream of nitrogen and phospholipids were then precipitated with ice-cold acetone (−20 °C for two hours). Acetone soluble lipids (ASLs) were further separated by high-performance liquid chromatography (Alliance 2690, Waters, Saint-Quentin-en-Yvelines, France) to collect pure mycolactone A/B and C fractions. ASLs were loaded into a reverse phase column (µBondapak C18 10 µm 3.9 × 300 mm, Waters) and eluted at 2 mL/min using an isocratic solvent composition: 20% water and 80% acetonitrile. Lipids were monitored at 363 nm (PDA 996, Waters) and eluting peaks corresponding to mycolactones A/B and C were individually collected in vials protected from light with aluminum foil. The leftover molecules were collected as mycolacone-free ASLs fraction. Semi-purified ASLs and purified mycolactones fractions were further characterized by LC/MS (Acquity iClass UHPLC and Vion IMS Qtof, Waters). Five µL of each fraction were eluted through a reverse phase column (BEH C18 1.7 µm 2.1 × 50 mm, Waters) using a linear gradient composed of water, acetonitrile and 0.1% formic acid. Lipids were ionized using a Z-spray electrospray source with the following parameters: positive mode, 3 kV capillary voltage, 40 V cone voltage. Ions were monitored with a High Definition Data Independent Acquisition method including ion mobility (HDMS (E), Waters) in order to collect parent and fragments masses according to the following settings: 50–1000 m/z, 0.1 s scan time, 19–40 eV collision energy ramp, mass calibration correction within run using a Lockmass (Leucine Enkephalin). UNIFI software (v. 1.8, Waters) was used to process raw data and collect components defined by: chromatography retention time, parent/fragments masses and ion mobility drift time. All collected components were screened for structures including mycolactones A/B, C, D, E, F and G (Chempsider, UK). Identified structures were validated using the following criteria: 1) H+ and/or Na+ adduct ions taken into account 2) <5 ppm mass error 3) >3 predicted fragments 4) <5 ppm mass error on parent isotopes (root mean square) and 5) <15% intensity deviation on parent isotopes (root mean square). The cytotoxic activity of semi-purified and purified mycolactones was assessed as previously described^20^. Briefly, L929 cells were cultured in Minimum Essential Medium (Gibco, Grand Island, USA) supplemented with 10% of heat inactivated foetal bovine serum (Gibco) and 1% of glutamine (Gibco). Cells were calibrated to 10^5^/mL and 270 µL were transferred to iCELLigence plates (Ozyme, Saint Quentin-en-Yvelines, France), plates were inserted to iCELLigence station for 24 h at 35 °C to allow cell adherence. After 24-hour incubation, plates were removed and 30 µL of mycolactones A/B and C, inactivated mycolactones A/B and C and 30 µL phosphate buffer saline (PBS) (negative control) were added. Plates were then inserted to iCELLigence station to monitor cell adherence for 24 h.

### Germination test

A 2-ml volume of spore suspension was dispensed into four sterile tubes. Then 20 µL of mycolactone AB/C were added to 3 out of the four tubes and 20 µL of PBS were added to the resting 2 mL spores’ suspension tube (negative control). At least 1,000 spores were observed in each tube and the number of germinated and non-germinated spores was recorded at time 0, 1 hour, 7 hours and 14 hours after incubation at 30 °C. This germination test was performed for the three species of fungi under investigation including *M. circinelloides*, *F. equiseti* and *S. apiospermum*.

### Chemoattractant effect assays

**Figure 1:**
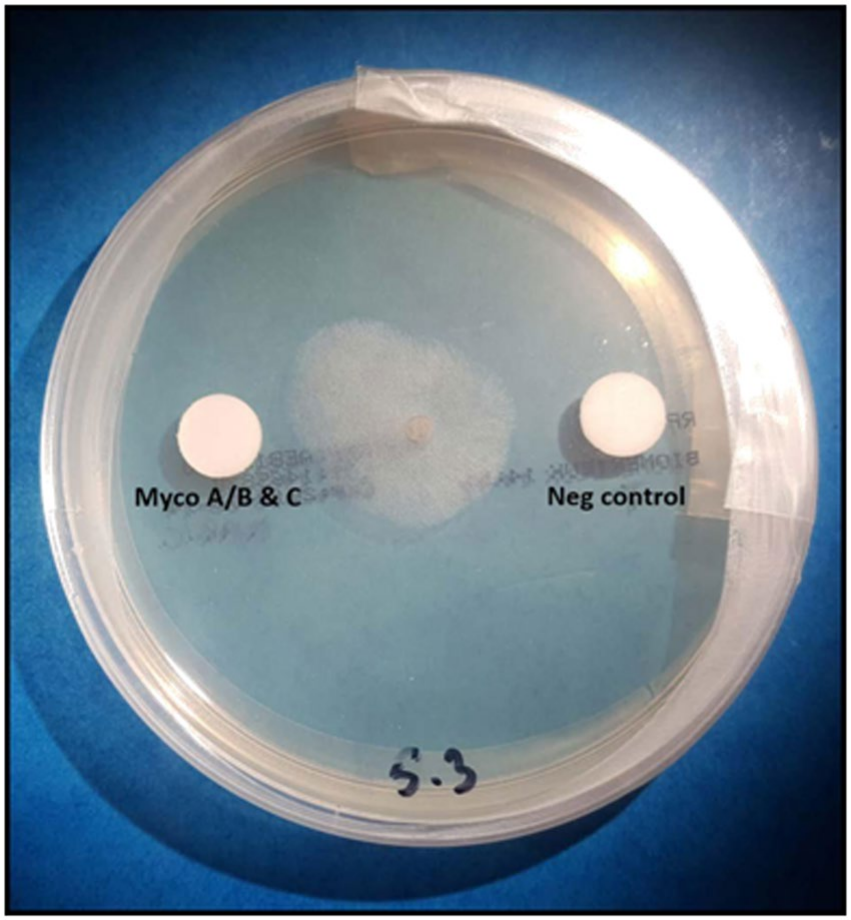
Assay showing the chemo-attractive effect of mycolactones A/B and C on a colony of *Mucor circinelloides*.

In a first step, three mm-diameter pieces of the colonies of each fungus were picked using a sterile biopsy punch (Kai Medical) and placed onto the center of a Roswell Park Memorial Institute (RPMI) agar medium plate (bioMérieux, Marcy l’Etoile, France). A PBS-impregnated filter paper disc (negative control) and an ASL impregnated disc were deposited on opposite sides at exactly 25 mm of the center of the colony (Fig. 1). This experiment was performed in sextuplicate for each fungal isolate tested. The RPMI agar media plates were then incubated for 24 hours at 30 °C. After incubation, a digital picture was taken for each plate and the millimetric distances between the edge of the fungal front and the edge of the disc were measured using Image J software^21^.

In a second step, fungal strains attracted by ASL in the above experiment were further subjected to similar experiments but with different types of molecules or mixtures thereof (i.e., mycolactone A/B, mycolactone C, mycolactone A/B and C and ASL) in eighteen replicates.

In a third step to confirm the attractant effect, the fungal strains attracted by ASL in the above experiment, were subjected to another test as described below. RPMI agar medium plates were cut in shape of the letter T (thus we called this test “the T-test”). A three mm-diameter piece of the colony of *M. circinelloides* was picked using a sterile biopsy punch (Kai Medical) and placed at the extremities of the vertical bar of the T-shape agar. A PBS-impregnated filter paper disc (negative control) and a disc impregnated with mycolactones A/B and C were deposited on the opposite extremities of the T-bar of the T-shape agar. This experiment was performed in eighteenplicates. The RPMI agar media were then incubated for 72 hours at 30 °C. After incubation the smallest distance between the front of the fungi and the front of the discs were measured.

### Statistical analyses

The effect of mycolactone on the germination of the fungal conidia was tested in a multivariate analysis via the Genmod procedure with the SAS 9.4 statistical software by using a negative binomial distribution, adjusted on the effect of time (quantitative variable) and by using generalized estimating equations to account for the non-independence of repeated measures. The distances measured between the front of the colony and PBS or mycolactones-impregnated discs were compared using the Wilcoxon rank signed rank test. Statistical analysis was performed using the R-software (CRAN-R the R Project for Statistical Computing). All tests were two-sided and a p-value less than 0.05 was considered significant.

## Results

### The fungal spores’ germination activity of mycolactone

The percentage of germinated spores of *M. circinelloides* was respectively 0%, 0.36%, 2.1%, 2.14% at 0, 1, 7 and 14-hour incubation with the mixture of mycolactones A/B and C whereas the percentage of germinated spores of the negative control (incubated with PBS only) was respectively 0%, 0.1%, 0.69% and 0.8% (Table 1). The percentage of germinated spores of *F. equiseti* was respectively 0%, 0.7%, 14.27%, 18.73% at 0, 1, 7 and 14-hour incubation with the mixture of mycolactones A/B and C whereas the percentage of germinated spores of the negative control was respectively 0%, 0.2%, 5.3% and 9.1%. The percentage of germinated spores of *S. apiospermum* was respectively 0%, 0.56%, 37.66%, 66.06% at 0, 1, 7 and 14-hour incubation with the mixture of mycolactones A/B and C whereas the percentage of germinated spores of the negative control was respectively 0%, 0.8%, 29.6% and 54.8%. Multivariate analysis demonstrated that mycolactones significantly enhanced the germination of spores in these three fungal species (p < 0.0001).

**Table 1:** Percentage values of fungal spore germination tests.

	Germination of fungal spores (%)							
	Mycolactones AB/C				PBS			
Time (H)	0	1	7	14	0	1	7	14
*M. circinelloides*	0	0.36	2.1	2.14	0	0.1	0.69	0.8
*F. equiseti*	0	0.7	14.27	18.73	0	0.2	5.3	9.1
*S. apiospermum*	0	0.56	37.66	66.06	0	0.8	29.6	54.8
P value	P < 0.0001							

Time is given in hours (H). PBS is for phosphate buffered saline as negative control.

### Chemoattractant activity of mycolactones

LC/MS analysis revealed the presence of mycolactones A/B and C within the ASLs solution, and the structure screening displayed several compound isomers for each type of mycolactone. Comparing the exact distance between the edge of the *M. circinelloides, S. apiospermum* or *F. equiseti* fungal colony and the PBS or ASL impregnated discs indicated that ASLs significantly (p = 0.03) attracted *M. circinelloides* (Supplementary File 1) but not the two other fungal isolates. We then assessed that the attractive effect of ASLs without mycolactones A/B and C on *M. circinelloides* was non-significant compared to the negative controls. These results indicated that the significant attraction of *M. circinelloides* may be due to one or several mycolactones in the ASLs.

**Figure 2:**
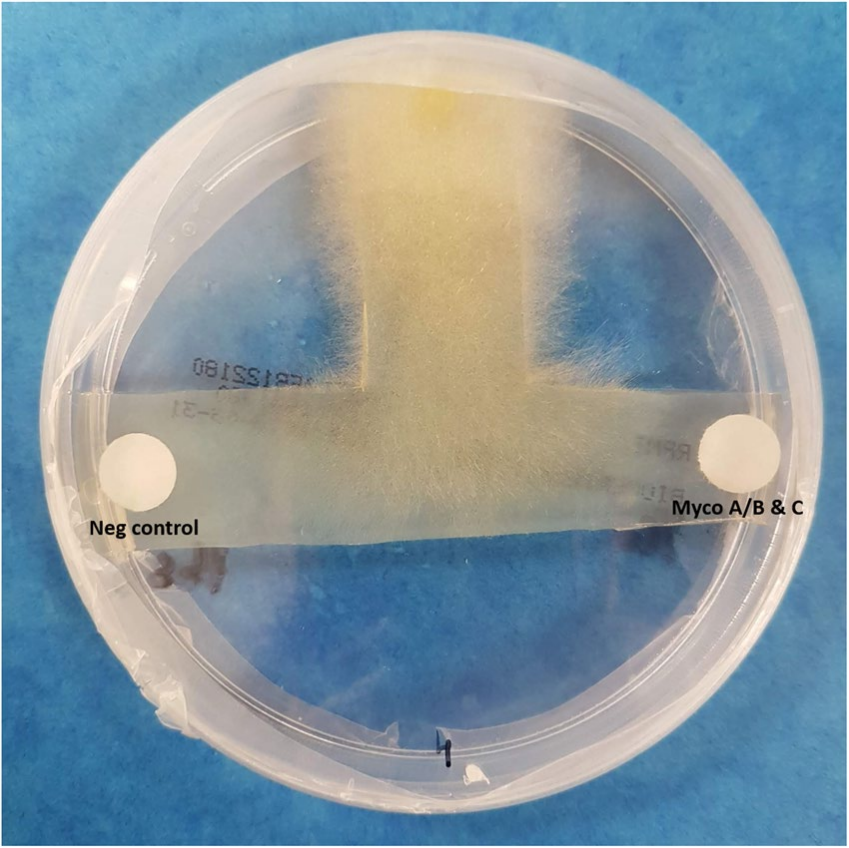
T test confirming the chemo-attractant effect of the mycolactone ABC mixture (P = 0.0046).

We further tested the hypothesis that *M. circinelloides* chemo-attraction was caused by mycolactones. Therefore, we tested the effect of mycolactone A/B alone, mycolactone C alone and a mixture of both mycolactones A/B and C using the procedure described above. We observed that a mixture of mycolactones A/B and C significantly attracted *M. circinelloides* (p = 0.03) (Fig. 1). A statistically non-significant (p = 0.22) chemo-attraction was also observed with using discs impregnated with mycolactone C alone (Supplementary File 1). No measurable attraction occurred in discs impregnated with only mycolactone A/B. These results indicated that the observed chemo-attraction of *M. circinelloides* was due to a mixture of mycolactones A/B and C and was not due to a distinct mycolactone. The effect of mycolactones A/B and C founded above was confirmed by the T-test showing that the mean distance between the mycolactones A/B and C impregnated disc and the growth front of *M. circinelloides* was significantly lower than the distance between the PBS impregnated disc and the front of the fungi (p = 0.0046) (Fig. 2).

## Discussion

Mycolactones secreted by environmental *M. ulcerans* and related *M. marinum* complex mycobacteria form a series of non-ribosomal synthesized macrolides^2^. In general, these macrolides synthesized by environmental Actinomycetes (which comprise mycobacteria) are known for their antibiotic and antifungal activities^22^, yet mycolactones in particular are only known as exotoxins inducing apoptosis of mammalian cells^23^. Therefore, we were surprised to observe that not only mycolactones did not exhibit anticipated antifungal activities but were activating fungal spores germination and had chemo-attractive effects on some fungi. Our observations were supported by the reproducibility of the results and the use of appropriate controls at each experimental step.

We observed that mycolactones enhanced *F. equiseti* spore germination. The same process was reported for myricetin, a flavonoid produced by tomato roots, on *F. oxysporum* spores^24^. Furthermore, the kinetic of *S. apiospermum* spore germination we observed was similar to that previously reported after exposure to some growth conditions^25^. Accordingly, asexual spore (conidia) germination is a complex process influenced by organic compounds in the rhizosphere, such as plant root flavonoids, which specifically stimulate the germination of *Fusarium* conidia^26^.

Furthermore, we observed a significant attraction of the fungus *M. circinelloides* to the mixture of mycolactones A/B and C. The observed attractive effect displayed a certain degree of isolate specificity. Indeed, of the three fungal species investigated using ASLs, only the *M. circinelloides* isolate was attracted whereas *S. apiospermum* and *F. equiseti* were not, the last tended to be repelled, albeit non-significantly. Interestingly, *M. circinelloides* belongs to the Zygomycetes family whereas *S. apiospermum* and *F. equiseti* are Septomycetes. We thought that differences of mycolactone effects between these two fungal groups were due to differences in the cell wall and/or cellular membrane. Indeed, these two structures play numerous and important roles including interaction with surrounding environment. Zygomycetes have coenocytic, about 6–16 µm-wide angle branched ribbon-like hyphae whereas Septomycetes hyphae are thin (2–3 µm) and regularly septate^27^. Our observations mirror previously reported demonstrations of positive chemotaxis of the motile *Pseudomonas fluorescens* to attractants composed of energy-rich organic compounds secreted by the soilborne plant pathogenic fungus *Macrophomina phaseolina*^28^. Mycolactones share structural similarities with strigolactones that are synthetized and released in the soil by plant roots to attract fungi in order to initiate symbiotic mycorrhizal association^29^. Strigolactones could also act as a repellant against phytopathogenic molds such as *Fusarium* species^30^. A cross-talk mediated by strigolactones has been demonstrated between *Mucor* species and *Arabidospsis thaliana*^31^. The attraction of *M. circinelloides* by mycolactones and their repellant effect on the fruit and crop pathogen *F. equiseti* that we observed were both remarkably similar to the effects of strigolactones in natural ecosystems.

**Figure 3:**
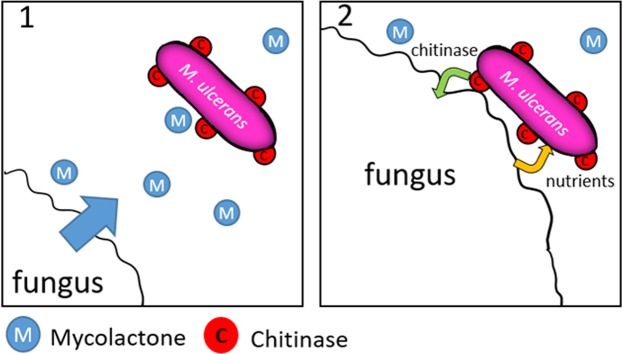
Potential interactions of *M. ulcerans* with fungi in its natural environment based on the conclusions of this work and the literature. (1) *M. ulcerans* lives in an ecological niche that comprises some fungi such as *Mucor circinelloides* and secrets mycolactones A/B and C, (2) Mycolactones A/B and C attract fungi towards non-motile *M. ulcerans*, its cell wall-bound chitinase may lyse some fungal cell wall chitin and liberate nutrients which may serve as a carbon source for *M. ulcerans*.

We thus speculated that *M. ulcerans* may use its secreted and diffusible mycolactones to attract motile environmental fungi, perhaps to feed on energy-rich organic compounds produced by fungal degradation pathways. This interaction with fungi may rely on the cell wall-bound chitinase encoded by *M. ulcerans*^15,32^ (Fig. 3). This would to confer mycolactone a new function distinct from cytotoxicity.

Further studies are needed using mycolactones and other fungi strains living in the same ecosystems as *M. ulcerans* in order to clarify the potential role of mycolactones in the natural habitats where *M. ulcerans* thrives. In particular, it has been suggested that *M. ulcerans* lives with complex microbial communities including bacteria and fungi^16^. However, the role of each microbial compartment and the relationships between the microbial communities remain unknown, although it has been proposed that the dynamics of *M. ulcerans* could result from complex interactions between environmental abiotic factors and variations in community assemblages^33^. Interestingly, it has been described that *Mycobacterium* genes are overrepresented in the rhizosphere, and these genes harbor a variety of nutrient transformation and degrading functions^34^. Further researches in this direction should explore the relationships between *M. ulcerans* and other microorganisms including fungi in targeted ecosystems as rhizosphere.

## Supplementary information

Dataset 1
